# Retrotransposition of Long Interspersed Nucleotide Element-1 Is Associated with Colitis but Not Tumors in a Murine Colitic Cancer Model

**DOI:** 10.1371/journal.pone.0116072

**Published:** 2015-02-24

**Authors:** Takeshi Otsubo, Tadashi Okamura, Teruki Hagiwara, Yukihito Ishizaka, Taeko Dohi, Yuki I. Kawamura

**Affiliations:** 1 Department of Gastroenterology, Research Center for Hepatitis and Immunology, Research Institute, National Center for Global Health and Medicine, Ichikawa 272-8516, Japan; 2 Section of Animal Models, Department of Infectious Diseases, Research Institute, National Center for Global Health and Medicine, 1-21-1, Toyama, Shinjuku-ku, 162-8655, Tokyo, Japan; 3 Department of Intractable Diseases, Research Institute, National Center for Global Health and Medicine, 1-21-1, Toyama, Shinjuku-ku, 162-8655, Tokyo, Japan; Massachusetts General Hospital, UNITED STATES

## Abstract

Long interspersed element-1 (L1) is a transposable element that can move within the genome, potentially leading to genome diversity and modified gene function. Although L1 activity in somatic cells is normally suppressed through DNA methylation, some L1s are activated in tumors including colorectal carcinoma. However, how L1-retrotransposition (L1-RTP) is involved in gastrointestinal disorders remains to be elucidated. We hypothesized that L1-RTP in somatic cells might contribute to colitis-associated cancer (CAC). To address this, we employed an experimental model of CAC using transgenic L1-reporter mice carrying a human L1-EGFP reporter gene. Mice were subjected to repeated cycles of colitis induced by administration of dextran sodium sulfate (DSS) in drinking water with injection of carcinogen azoxymethane (AOM). L1-RTP levels were measured by a quantitative polymerase chain reaction targeting the newly inserted reporter EGFP in various tissues and cell types, including samples obtained by laser microdissection and cell sorting with flow cytometry. DNA methylation levels of the human L1 promoter were analyzed by bisulfite pyrosequencing. AOM+DSS-treated mice exhibited significantly higher levels of L1-RTP in whole colon tissue during the acute phase of colitis when compared with control naïve mice. L1-RTP levels in whole colon tissue were positively correlated with the histological severity of colitis and degree of neutrophil infiltration into the lamina propria (LP), but not with tumor development in the colon. L1-RTP was enriched in LP mesenchymal cells rather than epithelial cells (ECs), myeloid, or lymphoid cells. DNA methylation levels of the human L1 promoter region showed a negative correlation with L1-RTP levels. L1-RTP was absent from most tumors found in 22-week-old mice. In conclusion, we demonstrated that L1-RTP was induced in the mouse CAC mucosa in accordance with the acute inflammatory response; however, retrotransposition appears not to have direct relevance to colitis-induced cancer initiation.

## Introduction

Long interspersed element -1 (L1) is a transposable element that can move within the genome to induce gene deletions, inversions, and insertions, potentially leading to genome diversity as well as altering gene function [1,2]. L1 is present in all mammals including humans and comprises approximately 17% of the human genome [3,4]. Although L1 activity is normally suppressed in somatic cells through epigenetic mechanisms, such as DNA methylation, some L1s are activated by environmental factors including carcinogens and proinflammatory compounds [5–8]. Importantly, L1 insertions were detected in several types of human cancers, including colorectal cancers [9–11]. Hypomethylation of the CpG islands at the L1 5’-UTR have been reported not only in cancers, but also in human inflammatory disorders [12,13] suggesting a potential role for L1 transposition in inflammatory conditions. This possibility is particularly important in gastrointestinal carcinogenesis, because chronic inflammation is one of the most important risk factors for gastrointestinal malignancy [14,15]. However, how L1-retrotransposition (L1-RTP) is involved in gastrointestinal inflammation and malignancy remains to be elucidated. We hypothesize that L1-RTP in somatic cells might contribute to colitis-associated cancer (CAC). To address this possibility, we employed an experimental model of CAC using the carcinogen azoxymethane (AOM) together with induction of colitis by dextran sulfate sodium (DSS) in the drinking water in conjunction with a transgenic mouse L1 reporter system. The mechanism of this CAC model has been extensively investigated using various types of gene-manipulated mice. For example, TNF-receptor 1 (TNFR1)-deficient mice showed decreased numbers of tumors, which were dependent upon deficiency of TNFR1 from their hematopoietic cells [16]. As a downstream signal of TNFR1, nuclear transcription factor kappa B (NF-kB) is reported to be critical in the inflammation-associated development of colitic cancer [17]. IL-6 also promotes tumor growth, possibly through activation of Janus-activated kinase (JAK) and the signal transducer and activator of transcription (STAT) 3 pathways [18–20].

In this study, we found that L1-RTP was positively associated with the severity of colitis. Activation of L1 was correlated with hypomethylation of CpG islands at the L1 5’-UTR. However, L1-RTP was not increased in the tumors in this model suggesting that L1-RTP is not significantly involved in tumor initiation in this CAC model.

## Materials and Methods

### Ethics statement

All experiments using mice were performed according to the Institutional Guidelines for the Care and Use of Laboratory Animals in Research with the approval of the Animal Care and Use Committee of the Research Institute, National Center for Global Health and Medicine (approval number 14039). Animals were euthanized with ketamine and xylazine and every effort was made to minimize suffering.

### Mice and the CAC model

**Fig 1:**
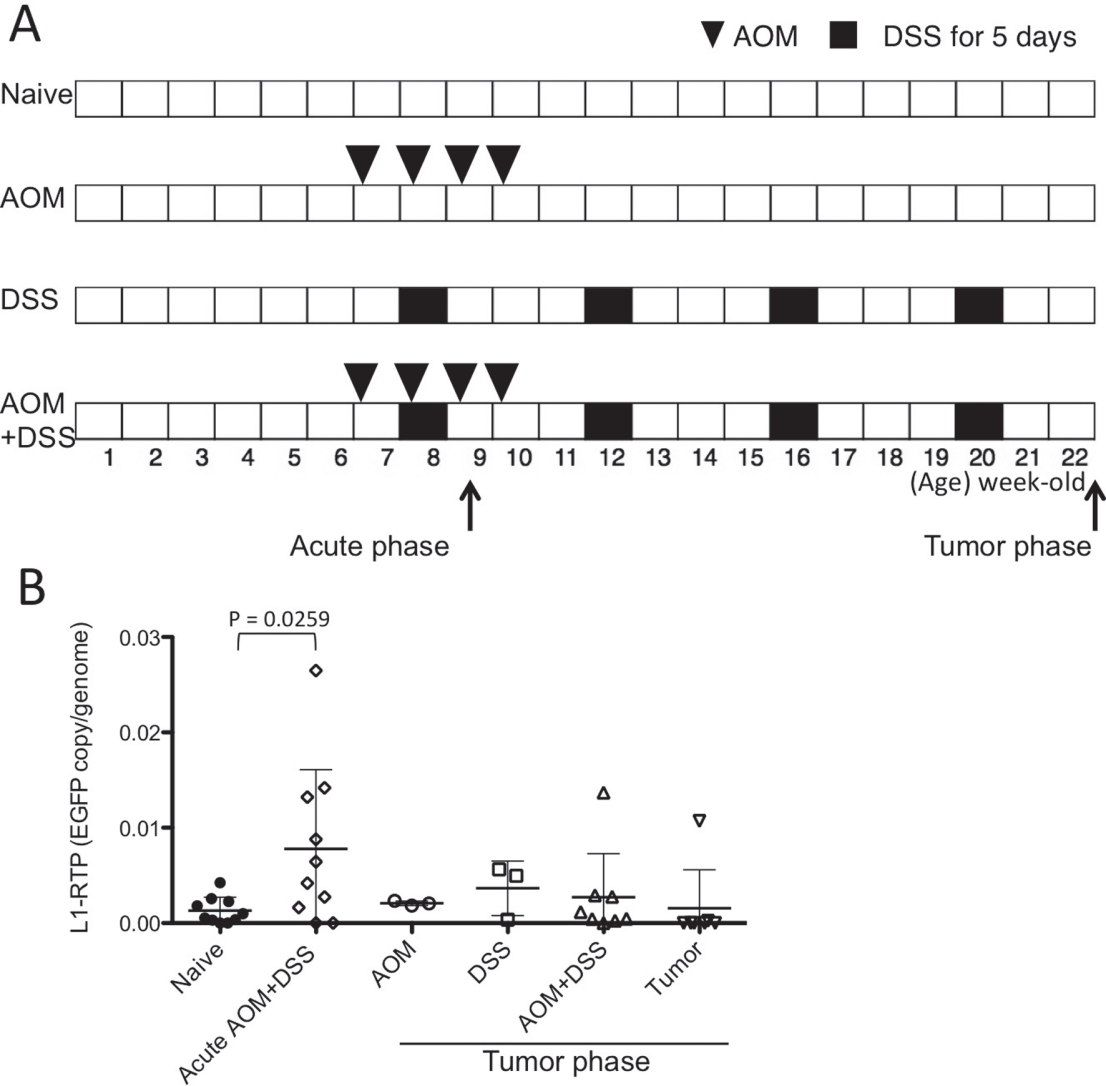
L1-RTP levels in AOM+DSS colitis mouse model. (**A**) Schematic time schedule of the AOM+DSS colitis mouse model. Arrows indicate the time-points associated with the acute inflammatory and tumor phases for harvesting the colon tissues and/or tumors. (**B**) L1-RTP levels were analyzed with genomic DNA extracted from whole distal colon tissues and/or tumors. The EGFP copy/genome was calculated by using ß-actin as internal control. Samples labeled “Acute AOM+DSS” were obtained during the acute phase in panel A, and others were collected during the tumor phase. AOM+DSS indicates residual colon tissue after removing visible tumors. Data were shown as mean ± SD. P values were calculated with an unpaired two-tailed t test.

A line of transgenic mice harboring a human L1-EGFP reporter gene (*hL1-EGFP*), named Line 4, was developed in our facility [5] and used in all experiments. Mice were kept under specific pathogen-free conditions during the experiments. CAC model protocols are shown in Fig. 1A. Weekly peritoneal injection of azoxymethane (AOM, 10 mg/kg, Sigma-Aldrich Japan) was started at 6 weeks of age and repeated four times. Mice were starved for the 12 h prior to AOM injection to enhance the effect of carcinogenesis with AOM [21]. Mice in the experimental group were given 3.5% (W/V) dextran sulfate sodium (DSS, Sigma-Aldrich Japan) in the drinking water for 5 days at 7 weeks of age, and this treatment was repeated four times on a monthly basis. At 22 weeks of age (tumor phase), colon tissues were obtained. Visible tumors were cut from the mucosa and separately analyzed from the background mucosa. In some experiments, colon tissues were obtained from 8-week-old mice following two injections of AOM and within seven days after stopping DSS treatment (acute phase).

### Quantification of L1-RTP

For the PCR assay, genomic DNA was prepared from the tissues and cells using a DNA extraction system (QuickGene; Fujifilm, Tokyo, Japan). The method for detection of the frequency of L1-RTP with a PCR targeting retrotranspositioned EGFP was described previously [22]. For analysis of tumors, one randomly selected tumor from the each mouse was subjected to DNA extraction and L1-RTP analysis.

### Histological analysis and laser microdissection

Whole colon tissues obtained during the acute inflammatory phase were rolled up and snap-frozen in liquid nitrogen. Frozen sections were fixed with cold acetone and stained with hematoxylin and eosin for histological analysis. Histological scores for the degree of crypt loss and cell infiltration were blindly assigned to each colon. Crypt loss was evaluated as the relative decrease in epithelial cell area in comparison with the naïve colon using a captured image analyzed by image J software (NIH, Bethesda, MD, USA): 0—no change or less than 10% decrease from naïve colon, 1—10–30% of epithelial cells were lost, 2—30–50%, 3—50–70%, 4—70–90%, 5—more than 90%. Cell infiltration scores: 0—no change from naïve colon, 1—focal infiltration limited to the mucosal layer, 2—focal infiltration in both mucosal and submucosal layers, 3—diffuse mucosal and submucosal infiltration. A score for each mouse was determined as the sum of the crypt score and the cell infiltration score. Neutrophil infiltration was determined by the nuclear morphology. In some experiments genomic DNA was obtained from frozen sections using a laser microdissection system (LMD 7000, Leica microsystems, Wetzlar, Germany) according to the manufacturer’s instructions.

### Cell separation

Epithelial cells (ECs) were isolated from the colon and treated with 10 mM EDTA in Hank’s Balanced Salt Solution (HBSS, Sigma Aldrich). Lamina propria (LP) cells (LPCs) were isolated using a Gentle MACS Dissociator (Milteny Biotech, Cologne, Germany) with the Lamina Propria Dissociation Kit for the mouse (Milteny Biotech). LPCs were stained with fluorescein isothiocyanate (FITC)-, R-phycoerythrin (PE)-, or allophycocyanin (APC)-labeled anti-CD3, anti-CD11b, anti-CD11c, anti-B220, anti-Gr-1, or anti-EpCAM antibodies (BD Biosciences, Franklin Lakes, NJ, USA), and 7-aminoactinomycin D to identify viable cells, and the cells were sorted with a flow cytometer (MoFlo, Beckman Coulter, Tokyo, Japan). Cell type sorting was performed with the following surface markers: T cells, EpCAM^-^CD3^+^CD11b^-^CD11c^-^; B cells including B220-low plasma cells, EpCAM^-^B220^+^CD11b^-^CD11c^-^; dendritic cells/macrophages, a mixture of EpCAM^-^CD3^-^CD11b^+^ cells, EpCAM^-^CD3^-^CD11c^+^ cells, and EpCAM^-^CD3^-^F4/80^+^ cells; granulocytes, EpCAM^-^Gr-1^high^ cells; non-epithelial non-hematopoietic cells, EpCAM^-^CD3^-^B220^-^CD11b^-^CD11c^-^F4/80^-^Gr-1^-^ cells.

### DNA methylation assay

To assess the methylation status of L1, bisulfite-pyrosequencing was performed with a PyroMark Q24 pyrosequencing machine (QIAGEN, Hilden, Germany) using pyro LINE kit (PyroMark Q24 CpG LINE-1 4 x 24, Cat. no.970042, QIAGEN).

### Statistical analysis

Statistical analysis was performed using the Prism 6 statistical program (GraphPad Software, Inc., La Jolla, CA, USA) with the methods indicated in the figure legends. For the analyses of difference unpaired one- or two-tailed Student’s t tests were used. Correlations were tested with two-tailed Pearson calculations. Values of P < 0.05 were considered significant.

## Results and Discussion

### L1-RTP levels increased in the acute phase of colitis

The experimental protocol for the CAC model is illustrated in Fig. 1A. Transgenic mice harboring a human L1-EGFP reporter gene (*hL1-EGFP*) [5] were used in all experiments. In this model, we successfully obtained colon tumors from 22-week-old mice after four injections of AOM and four cycles of DSS colitis. Three to twenty-four tumors were found in each colon (mean = 11.5, 11 mice). Untreated (naïve) or mice treated with only AOM or DSS did not develop colon tumors. To examine the induction of L1-RTP, we first obtained whole colon tissue from each group of mice during the tumor phase (22-week-old) or during the acute phase after the first administration of DSS (8-week-old). We initially attempted to detect cells expressing EGFP histologically or with flow cytometry; however, EGFP expression was not visible, probably because of low incidence of L1-RTP. Therefore, we quantified the frequency of L1-RTP with a PCR targeting retrotranspositioned EGFP as described previously [22]. As a result, we found that L1-RTP was significantly increased during the acute phase in the AOM+DSS colitis group, but not in tumors or in the background mucosa of tumor-bearing colons (Fig. 1B). Induction of L1-RTP was not seen in mice treated only with AOM or DSS. These results indicate that induction of L1-RTP is associated with acute colitis, but not maintained until the tumor phase, nor directly involved in tumorigenesis in this colitis-associated tumor model.

### L1-RTP levels correlate with the colitis severity but not with tumor numbers

**Fig 2:**
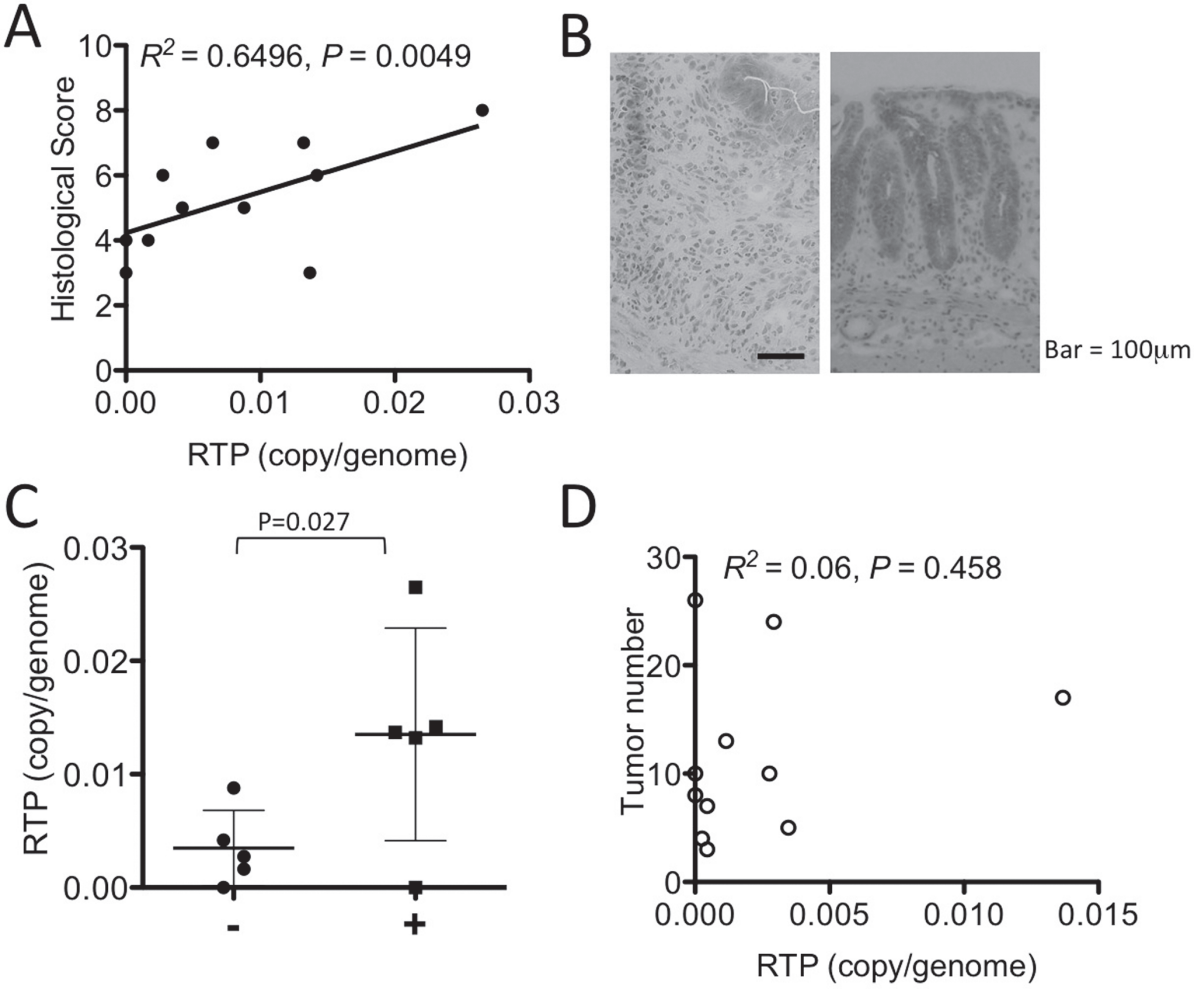
Correlation of the L1-RTP levels with histological scores, neutrophil infiltration, and tumor number. (A) The colitis histological score and L1-RTP levels in the whole distal colon during the acute phase mice showed a positive correlation. (B) Typical histological images of colitis with neutrophil infiltration (left) and without neutrophil infiltration (right) are shown. The bar represents 100 μm. (C) The L1-RTP levels were compared between the colon tissue without/with neutrophil infiltration into colon lamina propria during the acute and tumor phases. Data were shown as mean ± SD. The P value was calculated with an unpaired one-tailed t test. (D) The L1-RTP level of the background colonic mucosa and visible tumor numbers were not correlated.

Although L1-RTP was significantly induced during the acute phase of the CAC model, the levels of induction showed considerable individual differences (Fig. 1B). Therefore, we further examined the relation of L1-RTP with the severity of colitis. We found that histological scores, comprised of crypt loss and cell infiltration, were positively correlated with the frequency of L1-RTP (Fig. 2A). Because we observed that histologically severe inflammation was characterized by massive infiltration of granulocytes with a wide range of epithelial cell loss (Fig. 2B), samples were classified by the presence or absence of granulocyte infiltration. A trend toward higher L1-RTP was observed in the tissues with granulocyte infiltration than those without granulocytes (Fig. 2C). Although significant L1-RTP was not detected in tumors, we thought it possible that tumors might have grown by clonal expansion of L1-RTP negative cells, but occurrence of L1-RTP in the colonic background mucosa might have facilitated the development of tumors. Therefore, we examined the relationship between tumor numbers and the frequency of L1-RTP in the background mucosa; however, there was no significant correlation between these two factors (Fig. 2D). These results argue that L1-RTP is induced during acute colitis according to the severity of inflammation; however, the L1 in the colon is not maintained in an activated state from acute inflammation to the development of colon tumors after repeated bouts of colitis. Thus, L1-RTP does not appear to be directly involved in colon tumor initiation.

### L1-RTP was enriched in the colonic LP fraction in the acute phase of colitis

**Fig 3:**
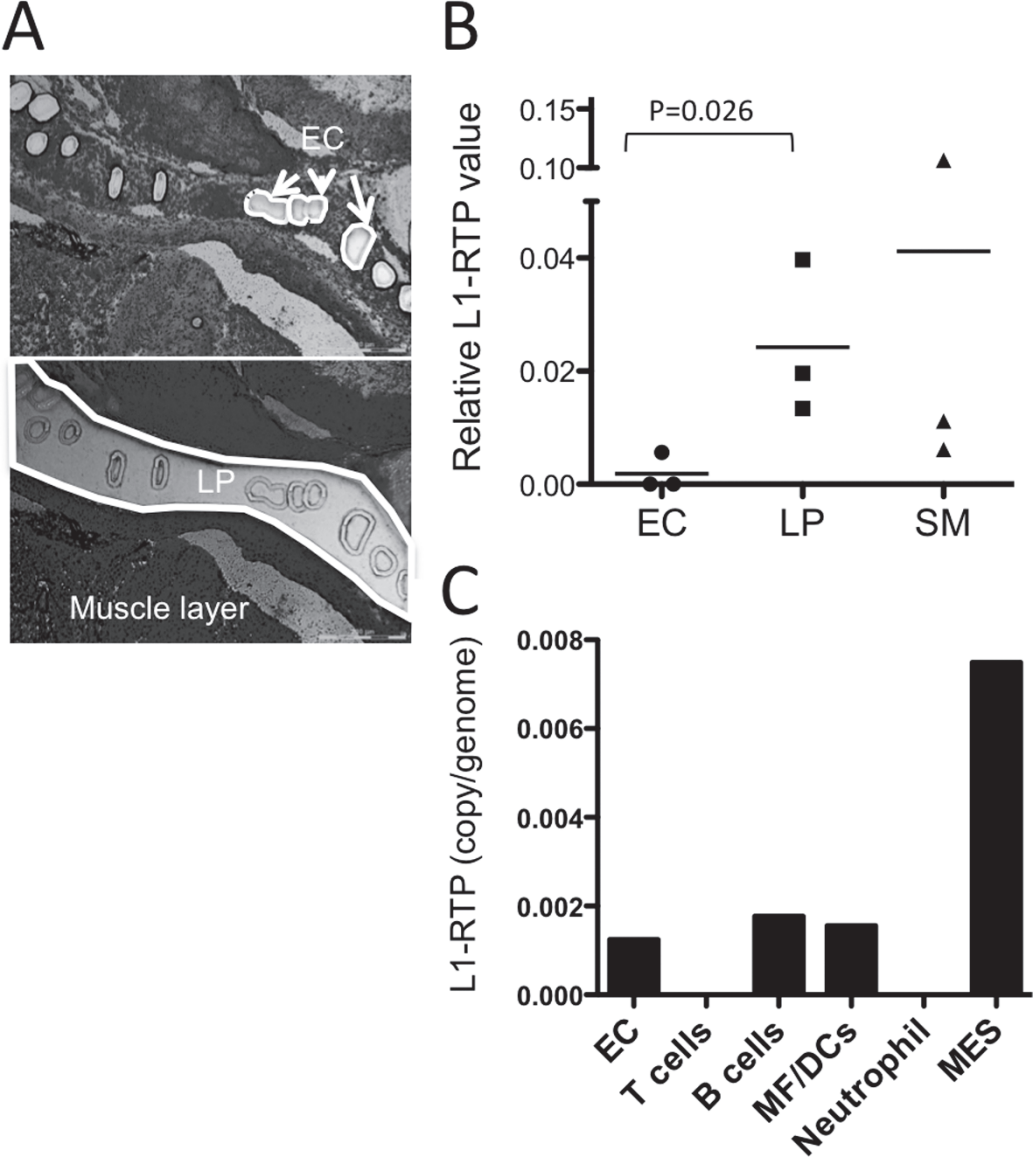
Cell type-specific L1-RTP levels in acute colitis mice. (A) Representative photograph of a section after microdissection for epithelial cells (EC, top), lamina propria (LP, bottom) and the remaining tissues (including submucosa and muscle layer) of colon section by laser microdissection. (B) L1-RTP levels were analyzed in colonic ECs, the LP, and the submucosa plus muscle layer (SM) isolated by microdissection from mice in the acute colitis phase. The relative L1-RTP value was normalized by using ß-actin as an internal control. Each plot indicates an individual mouse. P values were calculated with an unpaired one-tailed t test. (C) L1-RTP levels were examined in isolated ECs or sorted EpCAM negative-gated non-EC fractions by flow cytometry from mice in the acute colitis phase, as follows: T cells (EpCAM^-^CD3^+^), B cells (EpCAM^-^B220^+^), macrophage/dendritic cells (MΦ/DCs, EpCAM^-^CD11b^+^/CD11c^+^/F4/80^+^), Neutrophils (EpCAM^-^Gr^-^1^+^) and other cell types, most likely mesenchymal cells (MES, EpCAM^-^CD3^-^B220^-^CD11b^-^CD11c^-^F4/80^-^Gr-1^-^). Levels of L1-RTP from pooled cell fractions obtained from four mice are shown.

Next, we wanted to identify the cell types where L1-RTP took place in the colon. We separated epithelial and non-epithelial cells by microdissection of the mucosal layer of the colon obtained during the acute phase of inflammation. ECs were punched out, and then the remaining mucosal layer was dissected and subjected to DNA extraction (Fig. 3A). Unexpectedly, L1-RTP was enriched in the non-epithelial cell fraction of LP (Fig. 3B). Therefore, we next attempted to identify the cell types with L1-RTP by isolating LPCs with collagenase digestion and cell sorting using flow cytometry. As a result, L1-RTP was not detected in CD3^+^ T cells, B220^+^ B cells/plasma cells, CD11b^+^/CD11c^+^/F4-80^+^ macrophage/dendritic cells, or Gr1^+^ granulocytes (Fig. 3C). L1-RTP was high in the cell fraction, which was negative for these surface markers. Based on these results, mesenchymal type cells (MES) are most likely to be responsible for the increased L1-RTP during acute phase colitis. On the other hand, the frequency of L1-RTP in other cell types stayed at low levels. Further detailed analysis to identify the cell types inducing L1-RTP remains to be investigated.

### DNA methylation levels of the Tg-L1 promoter were inversely correlated with L1-RTP levels

**Fig 4:**
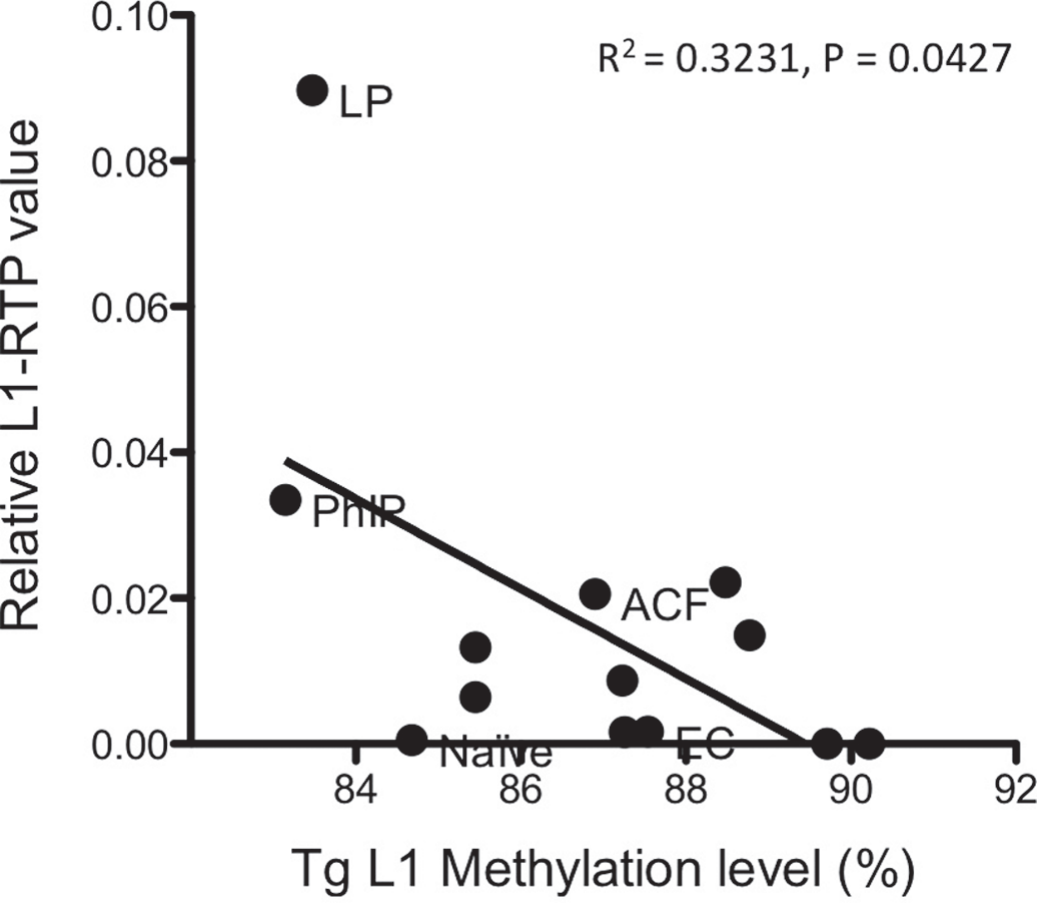
Correlation between DNA methylation levels of the human L1 promoter and L1-RTP levels. DNA methylation levels of the transgenic human L1 promoter were analyzed by bisulfite pyrosequencing with the primers targeting only the human L1 promoter sequence. Correlation between the levels of human L1 promoter DNA methylation and L1-RTP levels were analyzed using whole distal colon tissues with AOM+ acute DSS colitis (no label), isolated colonic ECs (EC) or LP (LP) fractions from AOM+DSS colitis, aberrant crypt foci (ACF) induced by AOM, whole colon of a naïve mouse, or the 2-Amino-1-methyl-6-phenylimidazo [4,5-b]pyridine (PhIP)-treated mice (a positive control of human L1-RTP reporter [8]). The P value was determined by a two-tailed Pearson’s calculation.

Since DNA methylation of the L1 promoter is known to prevent retrotransposition, we investigated the DNA methylation levels of the human L1 promoter in the transgenic mice. We selected various colon samples from naïve mice and mice with acute colitis plus AOM, aberrant crypt foci induced by four injections of AOM, ECs, and LPCs, together with 2-Amino-1-methyl-6-phenylimidazo [4,5-b] pyridine (PhIP)-treated mice (a positive control for the human L1-RTP reporter [8]), and measured DNA methylation levels of the CpG island associated with human L1 using bisulfite pyrosequencing of the 5’UTR region of the transgene. As expected, DNA methylation levels were generally high (>80%) in all samples. However, a statistically significant negative correlation was observed between DNA methylation levels and L1-RTP (Fig. 4) in these samples. Unexpectedly, methylation levels of naïve L1-RTP mice were not the highest among these samples, suggesting diverse levels of DNA methylation of the hL1 promoter in individual naïve mice.

This is the first report in which L1-RTP was directly demonstrated *in vivo*, induced by administration of a carcinogen with induction of colitis. In this study of CAC, we show that L1-RTP is not induced in epithelial cells or hematopoietic cells, but most likely in a mesenchymal cell type. Furthermore, we successfully measured DNA methylation levels of the human L1 promoter in this mouse model, and found a negative correlation between L1-RTP and DNA methylation of its promoter. This result supports the notion that hypomethylation of L1 is associated with L1-activation. On the other hand, we did not find L1-RTP in colon tumors or their background mucosa in this murine colon cancer model, although a correlation has been established between L1-RTP and cancer in humans as described in a recent review [23]. Instead, L1-RTP was associated with acute inflammation. Positive correlation of L1-RTP levels with the histological scores and the degree of cell infiltration suggests a potential contribution of L1-RTP to the severity of the acute colitis. In this sense, L1-RTP may be involved in the mechanism of exacerbation of inflammation. Our initial speculation was that the frequency of L1-RTP would be increased over the course of inflammation-associated tumor development. However, our overall results indicate that L1-RTP has a limited direct impact on tumorigenesis. Possible reasons for this unexpected result are as follows. First, our *in vivo* system does not detect the endogenous mouse L1-RTP, although detection of the RTP of the human L1 transgene was clear. Levels of human L1-RTP in the colon were generally low, and this detection sensitivity may not exactly represent the endogenous retrotransposition in the mice, which might affect the frequency of CAC. However, the fact that we detected increased L1-RTP in acute colitis but not in tumors indicates that the impact of L1-RTP on tumorigenesis is at least indirect. Second, previous study reported that L1 retrotransposition was present in preneoplastic liver but not in the subsequent liver adenocarcinoma [24]. The authors argued that tumor progression might have selected for the loss of the chromosome containing the active L1. In case of CAC, occurrence of chromosomal loss in tumors might also decrease the frequency of L1-RTP in tumors. Lastly, in this mouse model system, tumor expansion is limited to the mucosal layer of the colon, and tumors do not proceed to advanced stages like human cancers that show malignant features of invasion and metastasis. Therefore, this may not be a suitable model for investigation of the advanced stage of malignant cancers. Thus, our results indicate that the impact of L1-RTP on the initiation of colon cancer is limited in this CAC model; however, possible roles of L1-RTP in tumor progression and metastasis remain undetermined. To evaluate the potential involvement of L1-RTP in advanced stage cancer would require establishing different models of advanced cancer such as malignant tumor cell lines by inducing mutations in oncogenes or anti-oncogenes in these transgenic mice.

Recently, the role of mesenchymal stromal cells in various inflammatory conditions including colitis has been reported. The reparative and anti-inflammatory properties of mesenchymal stromal cells have been tested in a variety of animal models and have been applied in specific clinical settings [25]. Particularly, in AOM+DSS induced colitis model, bone marrow derived mesenchymal stem cells were shown to have anti-carcinogenic properties through transforming growth factor-ß signaling [26]. These results suggest the possibility that L1-RTP, which we found in mesenchymal cells, may disturb anti-inflammatory cell function. Further investigation of mesenchymal cells as a target cell type of retrotransposition will be interesting and may elucidate their potential role to influence colitis severity in the early phase of CAC.
